# Effects of Feeding Different Postbiotics Produced by *Lactobacillus plantarum* on Growth Performance, Carcass Yield, Intestinal Morphology, Gut Microbiota Composition, Immune Status, and Growth Gene Expression in Broilers under Heat Stress

**DOI:** 10.3390/ani9090644

**Published:** 2019-09-02

**Authors:** Ali Merzza Humam, Teck Chwen Loh, Hooi Ling Foo, Anjas Asmara Samsudin, Noordin Mohamed Mustapha, Idrus Zulkifli, Wan Ibrahim Izuddin

**Affiliations:** 1 Department of Animal Science, Faculty of Agriculture, Universiti Putra Malaysia, UPM Serdang 43400, Selangor, Malaysia; 2 Department of Animal Production, Faculty of Agriculture, University of Baghdad, Baghdad 10071, Iraq; 3 Institutes of Tropical Agriculture and Food Security, Universiti Putra Malaysia, UPM Serdang 43400, Selangor, Malaysia; 4 Department of Bioprocess Technology, Faculty of Biotechnology and Biomolecular Science, Universiti Putra Malaysia, UPM Serdang 43400, Selangor, Malaysia; 5 Institute of Bioscience, Universiti Putra Malaysia, UPM Serdang 43400, Selangor, Malaysia; 6 Department of Veterinary Pathology & Microbiology, Faculty of Veterinary Medicine, Universiti Putra Malaysia, UPM Serdang 43400, Selangor, Malaysia

**Keywords:** broilers, heat stress, antibiotic, postbiotic, growth performance, intestinal morphology, gut microbiota, immune status, gene expression

## Abstract

**Simple Summary:**

Heat stress is a serious issue in commercial broiler production in hot and humid countries, including Malaysia. Exposure of broilers to heat stress affects their health and productivity. In this context, antibiotics are widely used at sub-therapeutic levels as growth promoters to reduce stress and infectious diseases in order to sustain productivity in commercial broiler farms. However, the extensive use of antibiotics as growth promoters for a long time leads to the development of antibiotic-resistant bacteria and the possibility of antibiotic-resistant genes being transferred among organisms. Recently, postbiotics produced by *Lactobacillus plantarum* have been widely studied as a feed additive in order to replace in-feed antibiotics. However, to date, no studies have investigated the role of postbiotics in feed for broilers under heat stress.

**Abstract:**

The effects of feeding different postbiotics on growth performance, carcass yield, intestinal morphology, gut microbiota, immune status, and growth hormone receptor (GHR) and insulin-like growth factor 1 (IGF-1) gene expression in broilers under heat stress were assessed in this study. A total of 252 one-day-old male broiler chicks (Cobb 500) were randomly assigned in cages in identical environmentally controlled chambers. During the starter period from 1 to 21 days, all the birds were fed the same basal diet. On day 22, the birds were weighed and randomly divided into six treatment groups and exposed to cyclic high temperature at 36 ± 1 °C for 3 h per day from 11:00 to 14:00 until the end of the experiment. From day 22 to 42 (finisher period), an equal number of birds were subjected to one of the following diets: NC (negative control) basal diet; PC (positive control) basal diet + 0.02% oxytetracycline; or AA (ascorbic acid) basal diet + 0.02% ascorbic acid. The other three groups (RI11, RS5 and UL4) were basal diet + 0.3% different postbiotics (produced from different *Lactobacillus plantarum* strains, and defined as RI11, RS5 and UL4, respectively). The results demonstrated that birds fed RI11 diets had significantly higher final body weight, total weight gain and average daily gain than the birds that received the NC, PC and AA treatments. The feed conversion ratio was significantly higher in the RI11 group compared with the other groups. Carcass parameters were not affected by the postbiotic-supplemented diet. Postbiotic supplementation improved villi height significantly in the duodenum, jejunum and ileum compared to the NC, PC and AA treatments. The crypt depth of the duodenum and ileum was significantly higher in NC group compared to other treatment groups except RI11 in duodenum, and UL4 in ileum was not different with NC groups. The villus height to crypt depth ratio of duodenum and ileum was significantly higher for the postbiotic treatment groups and AA than the PC and NC treatment groups. The postbiotic RI11 group recorded significantly higher caecum total bacteria and *Lactobacillus* count and lower *Salmonella* count compared to the NC and PC treatment groups. The *Bifidobacterium* population in the NC group was significantly lower compared to the other treatment groups. The postbiotic (RI11, RS5 and UL4) and AA treatment groups showed lower Enterobacteriaceae and *E. coli* counts and caecal pH than the NC and PC treatment groups. The plasma immunoglobulin M (IgM) level was significantly higher in the birds receiving postbiotic RI11 than those receiving other treatments. The plasma immunoglobulin G (IgG) level was higher in the RI11 treatment group than in the NC, AA and RS5 groups. The plasma immunoglobulin A (IgA) level was not affected by postbiotic supplements. The hepatic GHR mRNA expression level was significantly increased in birds fed postbiotics RI11, RS5 and UL4, AA and PC compared to the NC-fed birds. Postbiotic RI11 led to significantly higher hepatic IGF-1 mRNA expression level compared to the NC, PC, and AA treatments. Mortality was numerically lesser in the postbiotic treatment groups, but not significantly different among all the treatments. In conclusion, among the postbiotics applied in the current study as compared with NC, PC and AA, RI11 could be used as a potential alternative antibiotic growth promoter and anti-stress treatment in the poultry industry.

## 1. Introduction

Heat stress remains a major problem in poultry production systems, having adverse effects on animal health and productivity [1,2]. Heat stress causes economic losses in the poultry industry [3] based on its negative impact on viability, immunity and growth performance in broiler chickens [2,4,5,6,7]. Furthermore, birds’ physiology and behaviour changes in response to heat stress to achieve thermoregulation, which negatively affects productivity owing to lower feed intake and digestive capacity [8,9,10,11] and alteration of the intestinal mucosa and microbiota ecology [12]. To combat some of the adverse effects of heat stress on poultry, especially relating to health and growth performance, the inclusion of feed additives such as antibiotics in the diet at sub-therapeutic levels as growth promoters is a common practice. Feeding broilers under heat stress with antibiotics has been shown to alleviate the effects of heat stress and improve growth performance [13,14]. However, excessive and prolonged use of antibiotics in animal feeds has raised concerns regarding antibiotic residues in animal products and the development of antibiotic-resistant bacteria [15,16], which could affect the health of both poultry and humans. This has led to the banning of antibiotic use in animals in several countries [17,18].

Ascorbic acid as an antioxidant and health-promoting agent is an alternative to growth promoter antibiotics in broiler chickens [19,20] and potentially be advantageous under heat-stress conditions. Dietary antioxidants such as ascorbic acid have been shown to be useful in compensating for inadequate biosynthesis of ascorbic acid in broiler chickens under heat stress and mitigating the negative effects of heat stress [21]. Ascorbic acid supplementation in heat-stressed broilers has been shown to alleviate the reduction of growth and feed intake [22,23], improve growth, feed efficiency and carcass traits, reduce serum concentrations of corticosterone and malondialdehyde (lipid peroxidation) [24], and enhance meat quality [25].

To replace the use of antibiotics, probiotics have been used as feed additives in poultry to promote a healthy gut environment and improve growth performance [26,27,28]. However, it has been reported that probiotic bacteria may acquire and transfer antibiotic resistance genes between organisms [16,29,30,31]. Subsequently, postbiotics, which are metabolites of probiotics, have been used as feed additives in livestock as a potential replacement for antibiotics and probiotics. Postbiotics have the same mechanism of action and capacity as probiotics owing to the presence of secondary metabolites from probiotics but without a living cell [32]. The presence of antimicrobial metabolites, such as organic acids and bacteriocins, in postbiotics can reduce the gut pH and inhibit the proliferation of opportunistic pathogens in the feed and gut of animals [33]. Postbiotics obtained from *Lactobacillus plantarum* exhibit inhibitory action on various pathogenic bacteria, including *Listeria monocytogenes*, *Salmonella typhimurium*, *Escherichia coli* and vancomycin-resistant Enterococci [34,35,36,37]. It has been demonstrated that the application of postbiotics as a feed additive in livestock promotes the growth performance and health of broilers [38,39,40], layers [41,42] and piglets [43,44], as well as enhancing rumen fermentation and health in ruminants [45,46]. In broilers under normal condition, postbiotic supplementation improves growth performance and health by promoting the immune status and gut health through the improvement of intestinal villus and increased lactic acid bacteria population and reduction of Enterobacteriaceae population and faecal pH [32,38,39,40,47].

In the context of postbiotics, apart from their ability to promote a healthy gut environment, the potential antioxidant capacity of postbiotics obtained from *L. plantarum* has been found to be particularly strong under heat-stress conditions. *Lactobacillus* and particularly *L. plantarum* cultures have been shown to have high antioxidative activities [48,49]. However, there is scarce information on postbiotics as an antioxidant source, apart from their benefits in terms of health and growth performance. In heat-stressed broilers, probiotics have been shown to increase the hepatic antioxidant capacity [26,28,50] and postbiotics from *L. plantarum* are expected to provide similar benefits to those from probiotic bacteria. Despite the data showing the benefits of postbiotics in broilers under normal conditions, there is still a paucity of information on the effects of postbiotics under heat-stress conditions. This necessitates the elucidation of dietary postbiotics in broilers under heat-stress conditions, particularly on growth performance and health aspects, in comparison with antibiotics and ascorbic acid. Hence, the objective of this study was to examine the effects of feeding postbiotics produced from different strains of *L. plantarum* on growth performance, carcass yield, intestinal morphology, gut microbiota, immune status, and hepatic growth hormone receptor (GHR) and insulin-like growth factor 1 (IGF-1) gene expression levels in broilers under heat stress.

## 2. Materials and Methods

### 2.1. Microorganisms and Maintenance

The *Lactobacillus plantarum* strains (RI11, RS5 and UL4) were obtained from the Laboratory of Industrial Biotechnology, Department of Bioprocess Technology, Faculty of Biotechnology and Biomolecular Sciences, Universiti Putra Malaysia. The *L. plantarum* strains were differentiated from each other by DNA sequence analysis [51] and their ability to produce different amino acids [52]. The bacterial cultures were maintained and revived as described by Foo, et al. [53]. The bacterial cultures were preserved at −20 °C in de Man, Rogosa and Sharpe (MRS) medium (Merck, Darmstadt, Germany) supplemented with 20% (*v*/*v*) glycerol.

### 2.2. Preparation of Postbiotics from L. plantarum Strains

Active *L. plantarum* (RI11, RS5 and UL4) cells were washed once with sterile 0.85% (*w*/*v*) NaCl (Merck, Darmstadt, Germany) solution and adjusted to 10^9^ CFU/mL to be used as an inoculum. Working cultures of *L. plantarum* (RI11, RS5 and UL4) were prepared by inoculating 10% (*v*/*w*) 10^9^ CFU/mL active bacterial cells into MRS media and incubating at 30 °C for 10 h, followed by centrifugation (Benchtop Microfuge 20R, Beckman Coulter, Brea, California, USA) at 10,000× *g* and 4 °C for 15 min. The cell-free supernatant (CFS) was then collected by filtration through a cellulose acetate membrane (Sartorius Minisart, 0.22 µm, Gottingen, Germany) as described by Loh, et al. [54]. The CFS was stored at −20 °C until the feeding trial was conducted. The liquid postbiotics were sprayed on the feed during mixing.

### 2.3. Animals Housing and Experimental Design

The experiment was undertaken following the guidelines approved by the Institutional Animal Care and Use Committee of the Universiti Putra Malaysia, which ensures that the care and use of animals for scientific purposes is humane and ethical. The study was conducted at the Animal Research Centre, Institute of Tropical Agriculture and Food Security, Universiti Putra Malaysia. A total of 252 1-day-old male broiler chicks (Cobb 500, Arkansas, USA) were obtained from a commercial hatchery. The chicks were individually wing-banded on arrival at the farm. Chicks were randomly assigned in three-tier cages (120 × 120 × 45 cm, length × width × height) with wire mesh flooring in three identical environmentally controlled chambers (9.1 × 3.8 × 2.3 m, length × width × height). The rearing conditions were in line with commercial recommendations. The temperature of the chambers was set at 32 ± 1 °C on day 1, and after that gradually reduced to about 24 ± 1 °C by day 21. The average relative humidity during the experimental period ranged between 61 and 90%. Birds were provided with continuous fluorescent lighting throughout the experimental period. Feed (mash form) and drinking water were provided ad libitum. The chicks were given vitamins and amino acids in drinking water to prevent stress for the first 3 days. All birds were vaccinated with Newcastle disease and infectious bronchitis (ND-IB) live vaccine (Fort Dodge, Iowa, USA) by eye drop at 4 and 18 days of age. The infectious bursal disease vaccine (MyVac UPM93, Malaysia) was applied on day 9 by eye drop. During the starter period from 1 to 21 days old, all the birds were fed the same basal diet. On day 22, the birds were weighed and randomly divided into six treatment groups with seven replicates for each treatment group and six birds per replicate. Diets were formulated based on the requirements of the Cobb 500 Management Guide [55] using FeedLIVE software (Table 1). From day 22 to day 42 (finisher period), an equal number of birds were subjected to one of the following diets: NC (negative control) basal diet; PC (positive control) basal diet + 0.02% (*w*/*w*) oxytetracycline; AA (antioxidant control) basal diet + 0.02% (*w*/*w*) ascorbic acid. Three further groups (RI11, RS5 and UL4) were the basal diet + 0.3% (*v*/*w*) of the respective postbiotics. From day 22, all the birds were exposed to cyclic high temperature at 36 ± 1 °C for 3 h from 11:00 to 14:00 every day until the end of the experiment at day 42. The time required for the temperature to increase from 24 to 36 °C was approximately 45 min. After the heat challenge, the time required to decrease the temperature from 36 to 24 °C was 1 h 30 min.

### 2.4. Sample Collection

On day 42, approximately 2 h and 45 min after commencing heat exposure, 14 broilers were randomly selected from each dietary treatment group (two birds per cage) and slaughtered according to Halal procedure, as outlined in the Malaysian Standard [56]. Blood samples were collected into blood collection tubes (BD Vacutainer^®^, New Jersey, USA) containing the anticoagulant EDTA and stored on ice. Blood samples were centrifuged at 3500× *g* at 4 °C for 15 min. Plasma samples were transferred into 1.5 mL microcentrifuge tubes and stored at −80 °C for immunoglobulin M (IgM), immunoglobulin G (IgG) and immunoglobulin A (IgA) analyses. The caecum content was quickly collected, frozen in liquid nitrogen, and stored at –80 °C for bacterial quantification analysis. Three parts of the small intestine were to determine the villi height and crypt depth. A part of the liver tissue was collected immediately after slaughtering the chickens and fast frozen in liquid nitrogen, then stored at −80 °C for gene expression analysis. The parts of carcass and viscera weight were taken and expressed as relative percentages of the live weight.

### 2.5. Growth Performance Measurements and Mortality

The initial bodyweight of the birds was recorded on day 22, then the body weight was recorded weekly until day 42 and average weight gains were calculated accordingly. Feed intake (FI) was recorded from day 22 to day 42 and feed conversion ratios (FCR) (feed/gain) were calculated. Mortality was recorded when it occurred.

### 2.6. Carcass Characteristics

Seven birds from each treatment group were chosen at day 42 to determine the carcass characteristics. The birds were weighed, slaughtered and had feathers removed before carcass measurement. Carcass parts including the viscera, breast, leg, backbone, wing and abdominal fat were cut and weighed individually. The weights of the carcass parts and viscera were presented as a percentage calculated according to the following Equation (1):

Cut yield (%) = (weight of cut/live body weight) × 100(1)

### 2.7. Intestinal Morphology

Intestinal morphology analysis was carried out according to the method described by Choe, et al. [42]. About 5 cm of the mid-portion of the duodenum, jejunum and ileum was excised carefully and phosphate-buffered saline was used to wash the samples before storing in formalin (10%). Dehydration of the samples was done in an automated tissue processor (Leica ASP 3000, Wetzlar, Germany) for 16 h before using a paraffin-embedding system (Leica RM 2155, Wetzlar, Germany) for sample embedment. A rotary microtome machine (Leica RM 2155, Wetzlar, Germany) was used to cut each sample at 4 μm and the sections were positioned on glass slides and heated at 57 °C to dry. The sections were stained using haematoxylin and eosin and viewed under a light microscope (Leica DM LB2, Wetzlar, Germany) with a fitted digital camera (Leica DFC 295, Wetzlar, Germany). For each six replicates, six villi sections were evaluated per slide per intestinal sample, thus giving a total of 36 measurements per sample. Villi height was measured from the tip of the villi to the villus crypt junction and crypt depth was defined as the depth of the invagination between two villi; both villi height and crypt depth were determined using Image-Pro Plus software as described by Touchette, et al. [57].

### 2.8. Caecum Microbial Population

Caecal content samples were collected immediately after slaughtering and kept at −80 °C for microbial quantification. The caecum microbes populations were determined according to the method described by Navidshad, et al. [58]. DNA was extracted from the caecal content samples using QIAamp^®^ DNA Stool Mini kits (Qiagen, Hilden, Germany). The DNA purification was done using a QIAamp spin column (Qiagen, Hilden, Germany) based on the kit instructions. Finally, the DNA was preserved at −20 °C until quantitative polymerase chain reaction (qPCR) analysis.

The DNA concentration and purity were determined by Nanodrop 2000 spectrophotometer (Thermo Scientific, Wilmington, DE, USA). Caecum microbial populations were determined by real-time polymerase chain reaction (RT-PCR). Caecum microbial contents were quantified based on the standard curve for the amplification of target microbes. A qPCR master mix (20 μL) was made using the QuantiNova™ SYBR Green PCR kit (Qiagen, Hilden, Germany), which comprised 10 μL of 2X SYBR Green Master Mix, 1 μL of each of 14 μM forward and reverse primers, 2 μL of DNA samples and 6 μL of RNase-free water. The targeted microbes and the sequences of the forward and reverse primers are presented in Table 2.

Real-time qPCR was carried out using the CFX96 real-time PCR system (BioRad, Hercules, California, USA) using the following conditions: initial heat activation at 94 °C for 2 min, followed by 40 cycles of denaturation for 10 s at 94 °C annealing for 30 s at 55 °C for total bacteria, 58 °C for *Lactobacillus*, 60 °C for *Bifidobacterium* and 50 °C for *Salmonella, E. coli*, *Enterococcus genus* and Enterobacteriaceae, and extension for 20 s at 72 °C. Melting curve analysis was carried out to confirm the specificity of amplification at the end of the amplification cycle.

### 2.9. Plasma Immunoglobulin Concentration

The IgG, IgM and IgA plasma concentrations were measured using chicken IgG, IgM and IgA ELISA kits (QAYEE-BIO, Shanghai, China) according to the manufacturer’s protocol. Briefly, 50 μL of standard and appropriately diluted sample (in duplicate) were loaded into microplate wells. Horseradish peroxidase (HRP, 50 μL) was mixed into each well of standard and sample, gently shaken, and then incubated for 60 min at 37 °C. After incubation, the well content was discarded and washed five times with washing solution. Chromogen solution A and B (50 μL of each) were added into each well and incubated at 37 °C for 10 min in the dark. Immediately after adding 50 μL of stop solution into each well, the absorbance was recorded at 450 nm using a microplate reader (BioTek™ ELx800™, Winooski, Vermont, USA). A blank containing standard solution (without sample or HRP) was measured and its absorbance subtracted from the absorbance for the samples and standard. The IgG, IgM and IgA plasma concentrations were obtained using standard curves for IgG, IgM and IgA.

### 2.10. RNA Extraction and RT-PCR of GHR and IGF-1 Genes

RNA was extracted from liver samples using a RNeasy^®^ Mini Kit (Qiagen, Hilden, Germany) following the manufacturer’s instructions. Liver samples (approximately 30 mg) were mixed with 600 μL of buffer RLT and centrifuged to collect the supernatant. An equal volume of 70% (*v*/*v*) ethanol was mixed with the supernatant. RNA collection and purification were conducted using RNeasy spin columns with buffer RW1, buffer RPE and lastly RNase-free water to elute the RNA. A nanodrop 2000 spectrophotometer (Thermo Scientific, Wilmington, DE, USA) was used to quantify the concentration and purity (260/280 nm ratio absorbance) of the extracted RNA. Purified RNA was converted into complementary DNA (cDNA) using a Quantitect^®^ reverse transcription kit (Qiagen, Hilden, Germany).

Real-time PCR was conducted using a Bio-Rad CFX96 PCR system (Bio-Rad Laboratories, Hercules, CA, USA). The housekeeping gene (GAPDH) was used to standardise the target genes. The qPCR master mix (20 μL) was made for every sample using a QuantiNova™ SYBR Green PCR kit (Qiagen, Hilden, Germany), which comprised 10 μL of 2X SYBR Green Master Mix, 1 μL of each of 14 μM forward and reverse primers, 2 μL of template cDNA and 6 μL of RNase-free water. The targeted gene primer sequences (forward and reverse) are shown in Table 3.

The qPCR cycling condition programme was performed as follows: initial denaturation temperature at 95 °C for 10 min, following by 40 cycles of denaturation at 95 °C for 15 s, annealing for 30 s at 57 °C for GADPH, IGF-1 and GHR genes, and finally 20 s of extension at 72 °C. Melting curve analysis was performed at the end of the amplification cycle to confirm the specificity of the amplification. The relative gene expression was measured based on the method described by Livak and Schmittgen [64]. The amplification efficiency of the housekeeping and target genes was established by conducting five-fold serial dilutions of cDNA as a standard curve.

### 2.11. Statistical Analysis

A completely randomized design was used in this study and all data analyses were conducted using the Statistical Analysis System software version 9.4 (SAS Institute, Cary, North Carolina, USA). The General Linear Model (GLM) procedure of the statistical software was applied and the means for dependent and independent variables (treatment groups and outcome) were compared using the Duncan Multiple Range Test. A *p*-value < 0.05 was considered significant for any statistical difference between the variables. A correlation test was conducted for caecal microbial population and pH. Mortality data were analysed by Chi-square test using Minitab 17 software.

## 3. Results

### 3.1. Growth Performance and Mortality

The indices included initial body weight (IBW), final body weight (FBW), cumulative body weight gain (CWG), average daily gain (ADG), cumulative feed intake (CFI) and FCR for the various treatment groups (Table 4). IBW, CFI and mortality were not affected (*p* > 0.05) by postbiotic supplementation compared with NC, PC and AA treatments. There was no significant difference in the FBW between the NC, PC, AA, RS5 and UL4 treatments. However, broilers fed with the RI11 postbiotic had significantly higher FBW as compared with the NC, PC and AA treatments. No significant difference was found among the postbiotics treatment groups. Postbiotic RI11 supplementation increased CWG and ADG in heat-stressed broilers (*p* < 0.05) in comparison with those fed the basal diet (NC), PC, AA and the other two postbiotics (RS5 and UL4). However, the other treatments did not improve the CWG and ADG of the broilers (*p* > 0.05). Compared with the NC group, broilers fed with various postbiotics exhibited a higher FCR (*p* < 0.05), whereas this effect was not observed with antibiotic or ascorbic acid supplementation (*p* > 0.05).

### 3.2. Carcass Yield

The postbiotic supplements had no effect (*p* > 0.05) on the carcass, breast, leg, wing, back, gizzard, spleen, fat and heart (Table 5). The carcass weight was significantly higher in the RI11 and UL4 birds than the other groups. However, there was no significant difference in carcass weight between postbiotic treatments (RI11, RS5 and UL4). On the other hand, the RS5 treatment group was not significantly different from the AA treatment group in terms of carcass weight. Similarly, there was no significant difference in carcass weight between the AA, PC and NC birds.

### 3.3. Intestinal Histomorphology

The villus height, crypt depth and villus height (VH): crypt depth (CD) ratio of the duodenum, jejunum and ileum of birds fed different postbiotic treatments under heat stress are shown in Table 6. Compared with the NC, supplementation with antibiotics (except duodenum), ascorbic acid and various postbiotics increased (*p* < 0.05) the villi height in the duodenum, jejunum and ileum of heat-stressed broilers, with postbiotics contributing higher (*p* < 0.05) effects than antibiotics and ascorbic acid. Postbiotic RI11 had a greater (*p* < 0.05) effect than the other two postbiotics. Broilers fed the PC, AA, RS5 and UL4 diets resulted in lower (*p* < 0.05) duodenal crypt depth. PC, AA, RI11 and RS5 diet had lower ileal crypt depth in comparison with the NC diet (*p* < 0.05). The broilers fed postbiotics RI11 and RS5 demonstrated significantly higher VH: CD ratios in the duodenum compared with the NC, PC and UL4 (except versus the AA group). Postbiotics RI11, RS5, and UL4 supplemented in broiler diets and the AA group significantly improved the VH: CD ratio in ileum compared to the NC and PC treatments, and in the comparison between the later groups, PC had a significantly higher VH: CD ratio than NC group. In the jejunum, the NC group had a significantly lower VH: CD ratio than the other treatment groups. However, there was no significant difference among all the treatments, except the NC group, which showed a significantly lower VH: CD ratio in the jejunum.

### 3.4. Caecum Microbial Population and pH

The caecal pH and caecal microbial populations for total bacteria, *Lactobacillus*, *Bifidobacterium*, Enterobacteriaceae, *E. coli*, *Enterococcus* and *Salmonella* in the broiler chickens fed the different postbiotics under heat stress are shown in Table 7. The results indicate that the groups fed RI11 and UL4 had significantly higher total bacteria compared to the NC and PC groups, but they were not significantly different as compared with RS5 and AA groups. However, the two latter groups were not different (*p* > 0.05) compared to the PC group. There was no significant difference between the PC and NC groups in total bacteria population. The RI11 group had a significantly higher *Lactobacillus* population compared to the other treatment groups, whereas the lowest population (*p* < 0.05) was found in the NC group, but there was no difference (*p* > 0.05) between the NC, PC and AA groups or between the UL4, RS5 and AA treatment groups. The *Bifidobacterium* population was the lowest (*p* < 0.05) in the NC group compared to the other treatment groups (AA, RI11, RS5 and UL4) and no significant difference was observed between the latter groups. The Enterobacteriaceae population was significantly higher in the NC group. In contrast, the Enterobacteriaceae population was significantly lower in the AA and RI11 groups as compared with the other treatment groups. However, there was no significant difference between the postbiotic and AA groups, between the RS5, UL4 and PC groups, or between the PC and NC groups for the Enterobacteriaceae population. The *E. coli* populations were significantly lower in the postbiotics and ascorbic acid diet groups compared with the PC and NC birds, but there was no difference (*p* > 0.05) between the latter groups. No effect was observed on the Enterococcus population (*p* > 0.05) among the treatment groups under heat stress (Table 7).

Postbiotic supplements affected the *Salmonella* population with RI11 having the lowest population (*p* < 0.05) compared to the other treatment groups. Nevertheless, no significant difference was observed in the *Salmonella* population between NC and PC, as well as between RI11, AA, RS5 and UL4. Caecal pH was lower (*p* < 0.05) in the RI11 and UL4 groups as compared with the NC and PC groups. However, there was no significant difference in caecal pH among postbiotic treatments (RI11, RS5 and UL4) and AA group. Similarly, the caecal pH was not significantly different between the RS5 and PC or PC and NC groups.

The test results for correlation between beneficial bacteria (Lactobacillus and Bifidobacterium) and caecal pH is shown in Table 8. A high negative correlation (*p* < 0.05) was found between beneficial bacteria (Lactobacillus and Bifidobacterium) and caecal pH. A negative correlation (*p* < 0.05) was also demonstrated between beneficial bacteria and Enterobacteriaceae, *E. coli* and *Salmonella*. However, there was a strong positive correlation (*p* < 0.05) between the pathogens and caecal pH.

### 3.5. Plasma IgG, IgM and IgA Concentration

**Figure 1:**
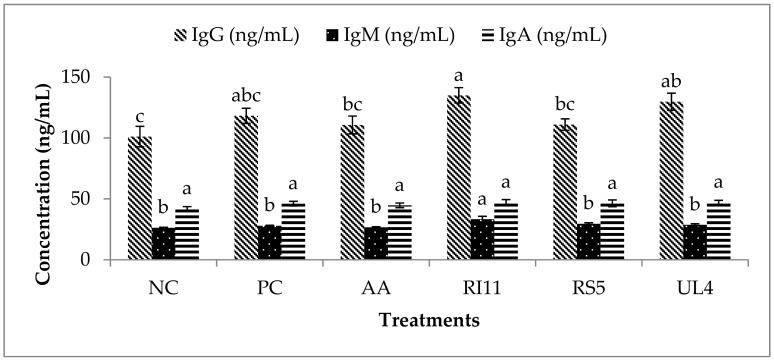
Plasma immunoglobulins in broiler chickens fed different postbiotics under heat stress. (**a**–**c**) Different letters on standard error bars indicate significant difference (*p* < 0.05). Data are shown as means and Standard error (n = 7).Treatments: Negative control (NC) = basal diet, positive control (PC) = basal diet + oxytetracycline 0.02% (*w*/*w*), AA = ascorbic acid 0.02% (*w*/*w*), RI11 = postbiotic RI11 0.3% (*v*/*w*), RS5 = postbiotic RS5 0.3% (*v*/*w*), UL4 = postbiotic UL4 0.3% (*v*/*w*).

The IgG, IgM and IgA plasma concentrations are presented in Figure 1. RI11 had a significantly higher concentration of IgM than the other treatment groups. No significant difference was observed for IgA among the treatment groups. In contrary, the IgG concentration was found to be higher (*p* < 0.05) in the RI11 group than the NC, AA and RS5 groups. However, there was no significant difference among RI11, UL4 and PC treatments. UL4 treatment gave a significantly higher concentration of IgG than NC treatment. There was no significant difference between RS5, AA, PC and NC for IgG.

### 3.6. Hepatic GHR and IGF-1 Gene Expression Levels

**Figure 2:**
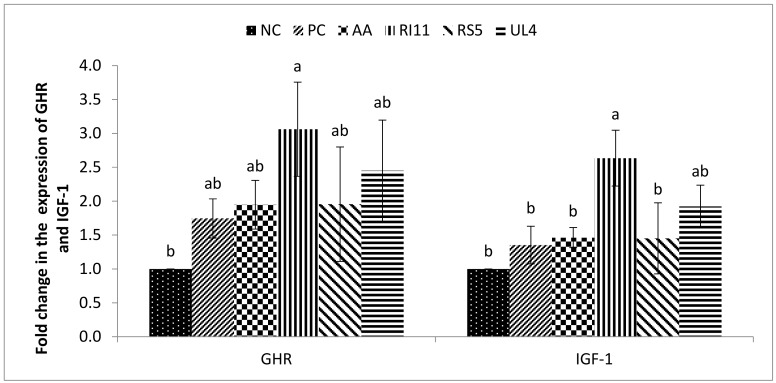
Hepatic GHR and IGF-1 mRNA expression level in broiler chickens fed different postbiotics under heat stress. a–c Different letters on standard error bars indicate significant difference (*p* < 0.05). Data are shown as means and standard error (n = 7). GHR = growth hormone receptor, IGF-1 = insulin-like growth factor 1. Treatments: Negative control (NC) = basal diet, positive control (PC) = basal diet + oxytetracycline 0.02% (*w*/*w*), AA = ascorbic acid 0.02% (*w*/*w*), RI11 = postbiotic RI11 0.3% (*v*/*w*), RS5 = postbiotic RS5 0.3% (*v*/*w*), UL4 = postbiotic UL4 0.3% (*v*/*w*).

Broilers supplemented with the postbiotic RI11 diet had significantly upregulated expression of IGF-1, followed by UL4 group (Figure 2). The GHR expression in the PC, AA, RS5, and UL4 treatment groups were not significantly different as compared with the NC treatment group, but the RI11 treatment group had significantly higher GHR expression than the NC group. However, the broilers fed the RI11 diet showed no significant difference in expression of the mRNA GHR gene compared to the PC, AA, RS5, and UL4 treatment groups. The RI11 group recorded significantly higher IGF-1 gene expression than the NC, PC, AA and RS5 treatment groups. However, no difference (*p* > 0.05) was observed among the latter treatment groups or between RI11 and UL4. The IGF-1 expression was not significantly different among the NC, PC, AA, RS5 and UL4 groups.

## 4. Discussion

### 4.1. Growth Performance, Mortality and Carcass Yield

Postbiotics have an impact on the growth rate of broiler chickens. Postbiotics have both bactericidal and bacteriostatic properties, which could decrease the multiplication of harmful bacteria in the gut. Postbiotics produced from *L. plantarum* have been reported to exhibit inhibitory effects on various pathogens [32,34,35,37]. In this study, the growth performance, such as FBW and FCR was significantly enhanced in the birds fed postbiotics as compared with the NC. Previously, better growth performance was obtained in birds receiving combinations of inulin and postbiotics [38]. This finding could be related to the postbiotics reducing the number of harmful microbes in the intestine, leading to the improved intestinal health and growth performance. Earlier studies also reported similar findings in broilers under normal conditions. For instance, the FBW and weight gain were higher in broilers receiving a mixture of postbiotics obtained from *L. plantarum* [32]. Another study also reported significantly improved growth performance in broiler chickens fed with a blend of *Lactobacillus* spp. culture and prebiotic [65]. Similarly, Kalavathy, et al. [66] reported an improvement in growth performance in broilers fed a mixture of 12 *Lactobacillus* strains. To relate our findings to other study conducted in poultry birds under heat stress, heat-stressed birds that were fed with dietary supplements containing *Lactobacillus* strains showed significant improvements in ADG and FI [59]. Another study showed significant improvements of body weight, weight gain, FI and FCR of broilers fed *Lactobacillus* strains compare with broilers fed oxytetracycline at a sub-therapeutic dose under heat stress [14]. Several studies have shown that probiotics improve the growth performance of broilers under heat stress by various actions, such as improvement of nutrient absorption [28,67]. As mentioned earlier, postbiotics have similar action to probiotics by promoting broiler growth performance, which may come from the enhancement of nutrient transporter gene expression [Na+-dependent glucose (SGLNC), galactose transporter (SGLRI11) and long-chain acyl CoA dehydrogenase genes] under heat stress, supported by Jahromi, et al. [28]. A later study conducted by Kalavathy, et al. [58] showed that increased utilisation of nutrients as a result upregulation of nutrient gene expression leads to improvement of the bodyweight of broilers. On the other hand, Rahimi and Khaksefidi [13] reported that there were no significant differences between antibiotic and probiotic groups in terms of broiler growth performance under heat-stress conditions. However, there is little information available regarding the effects of postbiotics on heat-stressed broilers. The administration of postbiotics to heat-stressed broilers in the current study was effective at promoting weight gain and feed conversion efficiency, in agreement with the beneficial effect of the probiotics found in heat-stressed broilers [68]. Hence, our results indicate that the postbiotics were effective at promoting the growth performance of broilers compared with the antibiotic group, despite the negative impact of heat stress.

In the present study the groups that received the postbiotics RI11, RS5 and UL4 had higher FCR values than the NC, PC and AA groups. Moreover, the RI11 birds had a higher FCR value among the postbiotic groups. This observation was consistent with reports where combinations of various postbiotics or postbiotics in combination with inulin enhanced feed efficiency in broiler chickens [32,38,39]. Although the AA birds were fed with the basal diet and ascorbic acid (antioxidant), the effect on FBW and FCR was not as great as that seen in those supplemented with postbiotics. Previous studies have reported contradicting findings for the effects of ascorbic acid on growth performance in heat-stressed birds [69]. The highest growth performance observed in layer birds under heat stress was in those fed with vitamin C compared with those fed betamine and vitamin E [70]. On the contrary, Jang, et al. [69] found no significant difference in growth performance following dietary supplementation with vitamin C, vitamin E and selenium in heat-stressed broiler chickens. Our results showed higher growth performance in the postbiotic groups compared to the ascorbic acid fed-group. The postbiotic groups showed a trend of lower mortality as compared with other treatments.

Dietary supplementation with different postbiotics had no effect on carcass yield in broiler chickens under heat stress. This outcome was similar to that reported by Kareem, et al. [71], who observed that the combination of postbiotics and inulin did not affect carcass yield in broilers. Furthermore, antibiotic and probiotic supplementation did not affect the carcass yield in broiler chickens [72]. Similarly, Abdel-Raheem and Abd-Allah [73] and Pelicano, et al. [74] reported that feeding of different probiotics had no significant effect on the carcass yield, leg, breast, heart, gizzard, spleen, wing, back and liver of broiler chickens at 42 days of age. In another study dietary supplementation with probiotics in broiler feed with environmental challenge had no effect on the yields of carcass, breast, leg and wings [75,76].

### 4.2. Intestinal Histomorphology

Villus height and crypt depth are important indicators of gut function and animal health [77]. The villi are the key components responsible for the absorbance of nutrients in the small intestine [78]. Increasing of villi height and decreased crypt depth may result in higher nutrient absorption, reduced secretion in the gastrointestinal tract and improvement of growth performance [79]. Fan, et al. [80] claimed that increased villus height and VH: CD ratio are positively correlated with increased epithelial cell turnover. Previously, it has been documented that postbiotic supplementation could improve intestinal morphology in broilers, as evidenced by increased villi height in the duodenum and ileum [38].

In this study, the broilers that received diets containing antibiotics, ascorbic acid and various postbiotics showed increased villi heights and VH: CD ratios in the duodenum (except those receiving antibiotics), jejunum and ileum as compared with the NC group. Broilers fed postbiotics and AA showed greater villi heights, VH: CD ratios and crypt depths than the PC group. Postbiotic RI11 contributed the greatest effect among the three postbiotics used in the current study. This finding provides further information to the previous studies showing positive effects of postbiotics on mucosal architecture with regards to villi height and improved growth performance [32,38]. Based on the findings herein, postbiotics induce their effect by improving the intestinal morphology and increasing the populations of beneficial bacteria, such as lactic acid bacteria [32,38,39,44],which reduces the risk of villi damage caused by lower pathogen populations in the gut, as found in the current study.

### 4.3. Caecum Microbial Population and pH

Intestinal microbiota is a vital determinant of gastrointestinal health [81]. Environmental stressors disturb the stability of the intestinal microbial ecology, resulting in dysbiosis [82]. Postbiotics have a beneficial effect by helping to maintain normal intestinal microbiota [38]. The present study evaluated whether such beneficial effect of postbiotics on caecal microbiota could be replicated under heat stress. The tested postbiotics had a significant effect on the specific caecal microbial population assessed in this study. Accordingly, the Enterobacteriaceae count was significantly reduced in all the broiler chickens supplemented with postbiotics compared to the NC and antibiotic groups. The present work also showed that the protective bacteria and total caecal bacteria count increased significantly in the heat-stressed broiler chickens fed with postbiotics compared with the NC group. Similarly, Kareem, et al. [38] found that broilers fed with combinations of a basal diet, inulin and varying concentrations of a postbiotic (RG14) had higher caecal total bacteria and *Bifidobacteria* compared with those fed basal diets and an antibiotic. Increased lactic acid bacteria populations have been reported following supplementation of broiler and layer feeds with four combinations of *L. plantarum*-derived metabolites [32,42]. The main reason for such an effect is that postbiotics have the capacity to promote the growth of protective bacteria (*Lactobacillus* and *Bifidobacterium*) while hindering the growth of pathogenic bacteria in the gut epithelium [83]. The effect is enhanced following a reduction in the intestinal and faecal pH induced by metabolites from *L. plantarum* [33]. The present study showed a high negative correlation between beneficial bacteria (*Lactobacillus* and *Bifidobacterium*) and caecal pH. A negative correlation was also found between beneficial bacteria and Enterobacteriaceae, *E. coli* and *Salmonella*. These relationships could be helpful in explaining the relationship between microbiota and caecal pH, as well as the inhibitory activity of beneficial bacteria against pathogens seen in the current study by lowering caecal pH. It has been reported that postbiotics contain bacteriocins, short-chain fatty acids (SCFAs), organic acids that result in the reduction of gut pH [32,44]. Bacterial species such as *E. coli* and *Salmonella* are intolerant to acidic environments, thus the actions of bacteriocins and SCFAs from postbiotics inhibit their activities. The physiology of these bacteria can be disrupted following the penetration of their cells by non-ionised organic acids [84]. However, the beneficial bacteria, such as *Lactobacilli* and *Bifidobacterium* favour a low-pH environment. Another role of these beneficial bacteria includes competition for intestinal adhesion sites and nutrients and eliciting immune responses [85]. These beneficial bacteria exert their protective action in the gut by competitive exclusion [86], preventing contact between pathogens and host epithelial cells [87]. This study showed that the beneficial effect of postbiotics on gut microbiota was enhanced in broilers even under heat-stress conditions. This was further evidenced in the heat-stressed broilers without postbiotic supplementation as increased viable counts of *Salmonella* and *E. coli* were observed. This is in accordance with an earlier report showing that heat stress may potentiate the increase in intestinal colonisation by pathogenic bacteria [59,88,89].

### 4.4. Plasma IgG, IgM and IgA Concentrations

Immunoglobulins play an essential role in immune regulation and mucosal defence; however, their functions can be affected by environmental stressors. IgM performs three main functions: the regulation of subsequent immune response, facilitating the production of IgG and the first immune response against foreign antigens [90]. IgA is crucial for protection at mucosal surfaces by preventing the entry, binding and colonisation of toxins and pathogens. By interacting with specific receptors and immune mediators, IgA influences variety of protective mechanisms [91].

The concentration of IgG was significantly greater in the RI11 postbiotic group compared with the NC, AA and RS5 supplemented groups. The postbiotic RI11 group had a higher plasma IgM concentration than all the other groups. This finding suggests that dietary postbiotic RI11 was more effective as compared with the other treatments in eliciting a humoral immune response in birds under heat stress. This result was consistent with the report by Kareem, et al. [38], where feeding a mixture of postbiotics and inulin had a positive effect on the humoral immune response in broiler chickens. In addition, our results provide further information on the humoral immune system of broilers when subjected to heat stress.

### 4.5. Hepatic IGF-1 and GHR Gene Expression

Hepatic IGF-1 influences the function of nutritional and growth hormones [92]. Growth hormone (GH) is released by the pituitary gland and stimulates the hepatic production of IGF-1 succeeding the actions of GH-activated GH receptors. Previous studies have shown that dietary postbiotics can influence the mRNA expression of IGF-1 and GHR in broiler livers [38]. Thus, our results corroborate those findings as the IGF-1 and GHR mRNA expressions were increased in the RI11 and UL4 birds under heat stress as compared with the NC group.

Specific mechanisms underline the local and systemic production of IGF-1. Microbiota was associated with the production of IGF-1 in an experiment conducted in mice [93]. Following colonisation with conventional microbiota, the expression of IGF-1 in the bone marrow was significantly increased, as well as the expression of Runx2, a target gene for IGF-1. The same experiment found increased production of adipose tissue IGF-1 and subsequent increased liver IGF-1 production in SCFA supplemented mice. Part of this event has been reported in chickens where a combination of prebiotics and *L. plantarum* led to increased faecal SCFA [38]. The intestinal microbiota increase production of SCFA when there is a higher population of beneficial bacteria components, such as *Lactobacilli* and *Bifidobacterium*. Such benefits have been found in several studies involving postbiotic supplementation in broilers [41]. Hence, the interplay between these events may contribute to the increase in circulating IGF-1 and expression of the GHR gene, as demonstrated in this study.

External stressors, such as heat stress, can affect the processes involved in the production of IGF-1 based on the negative impact on gut microbiota and antioxidant enzyme activities [94]. The heat-stressed birds without postbiotic supplementation in this study showed significantly lower expression of IGF-1. Hence, our results indicate that expression of IGF-1 can be applied as the basis for a growth index in heat-stressed broiler chickens.

## 5. Conclusions

In conclusion, supplementation with postbiotics, especially RI11, improved growth performance, intestinal morphology and immune response in broiler chickens under heat stress. Supplementation with various postbiotics enhanced the lactic acid bacteria count and intestinal villi height, and reduced the Enterobacteriaceae count. Higher body weight and weight gain and lower a FCR value in RI11-fed birds were accompanied by an increase in the expression levels of hepatic IGF-1 and GHR mRNA and plasma immunoglobulins (IgG and IgM) concentrations. Postbiotics, especially RI11, could be potential substitutes for antibiotics growth promoters and anti-stress agents in the poultry industry.

## Figures and Tables

**Table 1:** Composition and nutrient content of the finisher diets (day 22–42).

Ingredients%	Treatments Diets ^1^					
	NC	PC	AA	RI11	RS5	UL4
Corn	50	50	50	50	50	50
Soybean meal 48%	34.5	34.5	34.5	34.5	34.5	34.5
Palm oil	6.3	6.3	6.3	6.3	6.3	6.3
Wheat pollard	4	3.98	3.98	3.7	3.7	3.7
DCP 18% ^2^	2	2	2	2	2	2
Calcium carbonate	1.8	1.8	1.8	1.8	1.8	1.8
Choline chloride	0.1	0.1	0.1	0.1	0.1	0.1
Salt	0.3	0.3	0.3	0.3	0.3	0.3
DL-Methionine	0.32	0.32	0.32	0.32	0.32	0.32
L-Lysine	0.15	0.15	0.15	0.15	0.15	0.15
L-Threonine	0.05	0.05	0.05	0.05	0.05	0.05
Vitamin premix ^3^	0.15	0.15	0.15	0.15	0.15	0.15
Mineral premix ^4^	0.15	0.15	0.15	0.15	0.15	0.15
Antioxidant ^5^	0.08	0.08	0.08	0.08	0.08	0.08
Toxin binder ^6^	0.1	0.1	0.1	0.1	0.1	0.1
Oxytetracycline ^7^	0	0.02	0	0	0	0
Ascorbic acid ^8^	0	0	0.02	0	0	0
RI11	0	0	0	0.3	0	0
RS5	0	0	0	0	0.3	0
UL4	0	0	0	0	0	0.3
Total	100	100	100	100	100	100
Calculated nutrient level ^9^						
Metabolizable energy kcal/kg	3140.58	3140.57	3140.57	3140.57	3140.57	3140.57
Protein %	19.68	19.68	19.68	19.68	19.68	19.68
Fat %	8.319	8.319	8.319	8.319	8.319	8.319
Fiber %	3.973	3.973	3.973	3.973	3.973	3.973
Calcium %	1.097	1.097	1.097	1.097	1.097	1.097
Total phosphorus %	0.848	0.848	0.848	0.848	0.848	0.848
Available phosphorus for poultry %	0.49	0.49	0.49	0.49	0.49	0.49

^1^ NC (negative control) basal diet; PC (positive control) basal diet + 0.02% (*w*/*w*) oxytetracycline; AA (ascorbic acid) basal diet + 0.02% (*w*/*w*) ascorbic acid; RI11(basal diet + 0.3% (*v*/*w*) postbiotic RI11); RS5(basal diet + 0.3% (*v*/*w*) postbiotic RS5); UL4(basal diet + 0.3% (*v*/*w*) postbiotic UL4). ^2^ Dicalcium phosphate 18%. ^3^ Vitamin premix provided per kilogram of diet: Vitamin A 11494 IU; vitamin D_3_ 1725 IU; vitamin E 40 IU; vitamin K3 2.29 mg; cobalamin 0.05 mg; thiamine 1.43 mg; riboflavin 3.44 mg; folic acid 0.56 mg; biotin 0.05 mg; pantothenic acid 6.46 mg; niacin 40.17 mg; pyridoxine 2.29 mg. ^4^ Mineral mix provided per kilogram of diet: Co 0.6 mg, Cu 20 mg, Fe 100 mg, I 2 mg, Mn 110 mg, Se 0.2 mg, Zn 100 mg. ^5^ Antioxidant contains butylated hydroxyanisole (BHA). ^6^ Toxin binder contains natural hydrated sodium calcium aluminium silicates to reduce the exposure of feed to mycotoxins. ^7^ Oxytetracycline (200 mg/kg, purity ≥ 64.7%, Y.S.P. Industries (M) SDN BHD). ^8^ Ascorbic Acid (HmbG, Germany). ^9^ The diets were formulated using FeedLIVE International software (Nonthaburi, Thailand).

**Table 2:** The primer sequences of caecal targeting total bacteria, *Lactobacillus*, *Bifidobacterium, Enterococcus*, Enterobacteriaceae, *E. coli*, *Salmonella*.

Target Microbes	Primer Sequence 5′-3′	Product Size (bp)	References
Total bacteria	F—CGGCAACGAGCGCAACCC	145	[58]
	R—CCATTGTAGCACGTGTGTAGCC		
*Lactobacillus*	F—CATCCAGTGCAAACCTAAGAG	341	[59]
	R—GATCCGCTTGCCTTCGCA		
*Bifidobacterium*	F—GGGTGGTAATGCCGGATG	278	[60]
	R—TAAGCCATGGACTTTCACACC		
Enterococcus genus	F—CCCTTATTGTTAGTTGCCATCATT	144	[58]
	R—ACTCGTTGTACTTCCCATTGT		
Enterobacteriaceae	F—CATTGACGTTACCCGCAGAAGAAGC	195	[59]
	R—CTCTACGAGACTCAAGCTTGC		
*Escherichia coli*	F—GTGTGATATCTACCCGCTTCGC	82	[59]
	R—AGAACGCTTTGTGGTTAATCAGGA		
*Salmonella*	F—TCGTCATTCCATTACCTACC	119	[61]
	R—AAACGTTGAAAAACTGAGGA		

F = Forward, R = Reverse.

**Table 3:** The primer sequences of GHR, IGF-1, and GAPDH genes used for RT-qPCR.

Target Gene	Primer Sequence 5′-3′	Product Size (bp)	Reference
GHR	F—AACACAGATACCCAACAGCC	145	[62]
	R—AGAAGTCAGTGTTTGTCAGGG		
IGF-1	F—CACCTAAATCTGCACGCT	140	[62]
	R—CTTGTGGATGGCATGATCT		
GAPDH	F—CTGGCAAAGTCCAAGTGGTG	312	[63]
	R—AGCACCACCCTTCAGATGAG		

F = Forward, R = Reverse, GHR = Growth hormone receptor, IGF-1 = Insulin-like growth factor 1, and GAPDH = Glyceraldehyde-3-phosphate dehydrogenase.

**Table 4:** Body weight, body weight gain, feed intake, feed conversion ratio, and mortality in broiler chickens fed different postbiotics under heat stress.

Parameters	Dietary Treatments ^1^						SEM	*p*-Value
	NC	PC	AA	RI11	RS5	UL4		
IBW (g)	1020.31	1027.5	1001.29	1014	1004.48	1006.81	5.77	0.77
FBW (g)	2705.11 ^b^	2717.52 ^b^	2735.52 ^b^	2951.75 ^a^	2826.03 ^a,b^	2834.00 ^a,b^	18.92	0.001
CWG (g)	1704.21 ^b^	1746.54 ^b^	1759.34 ^b^	1944.94 ^a^	1802.77 ^b^	1805.39 ^b^	16.44	0.001
ADG (g)	81.15 ^b^	83.16 ^b^	83.77 ^b^	92.61 ^a^	85.84 ^b^	85.97 ^b^	0.78	0.001
CFI (g)	2932.1	3032.1	2970.8	3109.9	3004	3009	33.56	0.77
FCR (g/g)	1.72 ^a^	1.72 ^a^	1.70 ^a,b^	1.61 ^c^	1.68 ^b^	1.67 ^b^	0.007	0.001
Mortality	14/42	17/42	13/42	10/42	12/42	11/42	-	0.636

^a–c^ Means with different superscripts in the same row indicate significant difference (*p* < 0.05). ^1^ Dietary treatments: Negative control (NC) = basal diet, positive control (PC) = basal diet + oxytetracycline 0.02% (*w*/*w*), AA = ascorbic acid 0.02% (*w*/*w*), RI11 = postbiotic RI11 0.3% (*v*/*w*), RS5 = postbiotic RS5 0.3% (*v*/*w*), UL4 = postbiotic UL4 0.3% (*v*/*w*). SEM = Standard error of means. IBW = initial body weight. FBW = final body weight. CWG = cumulative weight gain. ADG = average daily gain CFI = cumulative feed intake. FCR = feed conversion ratio. Mortality presented as number of dead birds over total of the birds in each treatment.

**Table 5:** Carcass weight and carcass yield in broiler chickens fed different postbiotics under heat stress.

Parameters	Dietary Treatments ^1^						SEM	*p*-Value
	NC	PC	AA	RI11	RS5	UL4		
Carcass weight (g)	1905.9 ^c^	1956.9 ^c^	2059.6 ^b,c^	2350.4 ^a^	2222.3 ^a,b^	2306.7 ^a^	78.37	0.001
Carcass %	72.78	73.74	74.32	75.67	74.57	75.85	1.21	0.56
Breast %	27.11	28.51	27.07	28.82	26.75	28.33	0.98	0.56
Leg %	20.05	18.51	22.18	20.33	20.54	21.35	0.75	0.15
Wing %	7.24	7.52	8.04	7.61	7.53	7.68	0.27	0.59
Back %	18.12	16.81	18.02	18.39	19.35	17.88	0.74	0.33
Liver %	1.97	1.86	1.84	1.85	1.86	1.82	0.06	0.75
Gizzard %	2.16	2.15	2.02	1.96	1.91	1.82	0.13	0.35
Spleen %	0.08	0.09	0.06	0.08	0.07	0.06	0.01	0.62
Abdominal fat %	1.28	1.23	1.15	1.11	1.15	1.05	0.13	0.87
Heart %	0.38	0.4	0.43	0.4	0.4	0.38	0.03	0.88

^a–c^ Means with different superscripts in the same row indicate significant difference (*p* < 0.05). ^1^ Dietary treatments: Negative control (NC) = basal diet, positive control (PC) = basal diet + oxytetracycline 0.02% (*w*/*w*), AA = ascorbic acid 0.02% (*w*/*w*), RI11 = postbiotic RI11 0.3% (*v*/*w*), RS5 = postbiotic RS5 0.3% (*v*/*w*), UL4 = postbiotic UL4 0.3% (*v*/*w*). SEM = Standard error of means.

**Table 6:** Intestinal histology of broiler chickens fed different postbiotics under heat stress.

Parameters	Dietary Treatments ^1^						SEM	*p*-Value
	NC	PC	AA	RI11	RS5	UL4		
Villi height, μm								
Duodenum	1178.5 ^c^	1266.08 ^b,c^	1310.1 ^b^	1577.57 ^a^	1368.24 ^b^	1291.15 ^b^	20.99	<0.0001
Jejunum	893.71 ^d^	1000.45 ^c^	1058.58 ^b,c^	1117.61 ^a,b^	1142.34 ^a^	1183.97 ^a^	15.5	<0.0001
Ileum	611.43 ^d^	702.84 ^c^	839.08 ^b^	932.65 ^a^	886.69 ^a,b^	885.7 ^a,b^	17.11	<0.0001
Crypt depth, μm								
Duodenum	239.76 ^a^	195.05 ^b,c^	180.67 ^b,c^	211.18 ^a,b^	174.45 ^c^	204.54 ^b,c^	5.02	0.001
Jejunum	143.64	135.51	142	147.45	142.18	145.49	14.49	0.945
Ileum	127.55 ^a^	100.77 ^b^	104.00 ^b^	98.19 ^b^	102.40 ^b^	111.94 ^a,b^	2.59	0.007
Villi height: Crypt depth								
Duodenum	4.9 ^c^	6.60 ^b^	7.40 ^a,b^	7.94 ^a^	8.16 ^a^	6.71 ^b^	0.20	<0.0001
Jejunum	6.35 ^b^	7.82 ^a^	7.82 ^a^	7.72 ^a^	8.26 ^a^	8.29 ^a^	0.19	0.040
Ileum	4.95 ^c^	6.96 ^b^	8.45 ^a^	9.58 ^a^	8.71 ^a^	8.34 ^a^	0.26	<0.0001

^a–d^ Means with different superscripts in the same row indicate significant difference (*p* < 0.05). ^1^ Dietary treatments: Negative control (NC) = basal diet, positive control (PC) = basal diet + oxytetracycline 0.02% (*w*/*w*), AA = ascorbic acid 0.02% (*w*/*w*), RI11 = postbiotic RI11 0.3% (*v*/*w*), RS5 = postbiotic RS5 0.3% (*v*/*w*), UL4 = postbiotic UL4 0.3% (*v*/*w*). SEM = Standard error of means.

**Table 7:** Caecum microbial population (log_10_ CFU/g) and pH in broiler chickens fed different postbiotics under heat stress.

Parameters	Dietary Treatments ^1^						SEM	*p*-Value
	NC	PC	AA	RI11	RS5	UL4		
Total bacteria	9.65 ^c^	9.90b ^c^	10.18 ^a,b^	10.25 ^a^	10.13 ^a,b^	10.23 ^a^	0.04	0.004
*Lactobacillus*	7.79 ^c^	7.90b ^c^	8.07 ^b,c^	8.65 ^a^	8.26 ^b^	8.19 ^b^	0.06	0.002
*Bifidobacterium*	4.48 ^b^	5.87 ^a^	6.29 ^a^	6.56 ^a^	6.48 ^a^	6.46 ^a^	0.17	0.001
Enterobacteriaceae	7.57 ^a^	7.21 ^a,b^	6.35 ^c^	6.34 ^c^	6.70 ^b,c^	6.67 ^b,c^	0.11	0.001
*Escherichia coli*	7.14a ^b^	7.71 ^a^	6.91 ^b^	6.67 ^b^	6.82 ^b^	7.07 ^b^	0.09	0.008
*Enterococcus*	7.71	8.04	8.23	8.33	8.39	8.12	0.09	0.308
*Salmonella*	3.26 ^a^	2.77 ^a^	2.38 ^a,b^	1.77 ^b^	2.39 ^a,b^	2.46 ^a,b^	0.13	0.034
Caecal pH	6.14 ^a^	6.02 ^a,b^	5.91 ^b,c^	5.83 ^c^	5.88 ^b,c^	5.91 ^b,c^	0.03	0.009

^a–c^ Means with different superscripts in the same row indicate significant difference (*p* < 0.05). ^1^ Dietary treatments: Negative control (NC) = basal diet, positive control (PC) = basal diet + oxytetracycline 0.02% (*w*/*w*), AA = ascorbic acid 0.02% (*w*/*w*), RI11 = postbiotic RI11 0.3% (*v*/*w*), RS5 = postbiotic RS5 0.3% (*v*/*w*), UL4 = postbiotic UL4 0.3% (*v*/*w*). SEM = Standard error of means.

**Table 8:** Correlation test (R) between measured caecum microbial population and pH in broilers fed postbiotics, ascorbic acid and antibiotic under heat stress.

	*Lactobacillus*	*Bifidobacterium*	Enterobacteriaceae	*E. coli*	*Salmonella*	Caecum pH
*Lactobacillus*		0.34 *	−0.33 *	−0.38 *	−0.37 *	−0.50 ***
*Bifidobacterium*			−0.38 *	−0.23	−0.34 *	−0.55 ***
Enterobacteriaceae				0.69 ***	0.59 ***	0.45 **
*E. coli*					0.32 *	0.33 *
*Salmonella*						0.48 ***
Caecum pH						

* = significant at (*p* < 0.05). ** = significant at (*p* < 0.01). *** = significant at (*p* < 0.001). (n = 48 samples per microbe).
